# Comprehensive Method for Culturing Embryonic Dorsal Root Ganglion Neurons for Seahorse Extracellular Flux XF24 Analysis

**DOI:** 10.3389/fneur.2012.00175

**Published:** 2012-12-14

**Authors:** Miranda Lange, Yan Zeng, Andrew Knight, Anthony Windebank, Eugenia Trushina

**Affiliations:** ^1^Department of Neurology, Mayo ClinicRochester, MN, USA

**Keywords:** Seahorse XF24 Extracellular Flux analysis, mitochondrial respiration, oxygen consumption rate, extracellular acidification rate, embryonic dorsal root ganglion neurons

## Abstract

Changes in mitochondrial dynamics and function contribute to progression of multiple neurodegenerative diseases including peripheral neuropathies. The Seahorse Extracellular Flux XF24 analyzer provides a comprehensive assessment of the relative state of glycolytic and aerobic metabolism in live cells making this method instrumental in assessing mitochondrial function. One of the most important steps in the analysis of mitochondrial respiration using the Seahorse XF24 analyzer is plating a uniform monolayer of firmly attached cells. However, culturing of primary dorsal root ganglion (DRG) neurons is associated with multiple challenges, including their propensity to form clumps and detach from the culture plate. This could significantly interfere with proper analysis and interpretation of data. We have tested multiple cell culture parameters including coating substrates, culture medium, XF24 microplate plastics, and plating techniques in order to optimize plating conditions. Here we describe a highly reproducible method to obtain neuron-enriched monolayers of securely attached dissociated primary embryonic (E15) rat DRG neurons suitable for analysis with the Seahorse XF24 platform.

## Introduction

Genetic and environmental stressors associated with various neurodegenerative disorders alter the metabolic state of the cell causing a quick remodeling of catabolic and anabolic pathways in order to manage and/or adapt to the new environment. Bioenergetic deficits are often coupled with abnormal cellular pathologies and phenotypic changes, which exhibit unique profiles (Chen and Chan, 2006; Finsterer, 2006; Scaglia, 2010; Zheng et al., 2011; Saxena, 2012). Thus, the relative state of aerobic and glycolytic (anaerobic) metabolism is indicative of the overall health of the cell, and analysis of nutrient and biomolecular flow in live cells is essential to understanding the relevant cellular responses to disease pathology and/or changes in the extracellular environment. The Seahorse XF24 Extracellular Flux analyzer (hereafter referred to as the Seahorse XF24 analyzer) allows non-destructive, non-invasive, and sensitive analysis of the bioenergetic state of live intact cells in real-time providing invaluable assessments of mitochondrial function (Wu et al., 2007; Ferrick et al., 2008).

Recent data demonstrate that mitochondrial dysfunction is an early event underlying the development and progression of multiple neurodegenerative disorders including drug-induced peripheral neuropathies (Trushina and McMurray, 2007; Yao et al., 2009; Podratz et al., 2011; Xiao et al., 2011; Liu et al., 2012). In particular, altered mitochondrial fission, fusion, and motility were found in Charcot–Marie Tooth disease, as well as optical and diabetic neuropathies (Chen and Chan, 2006; Frank, 2006; Chowdhury et al., 2012; Saxena, 2012; Schapira, 2012). These are novel observations with limited mechanistic understanding. Cultures of adult and embryonic dorsal root ganglion (DRG) neurons represent the most relevant *in vitro* model to study peripheral sensory neuropathies (Melli and Hoke, 2009), and utilization of the Seahorse XF24 analyzer could significantly add to the understanding of disease mechanisms in order to find efficient therapeutic approaches.

Contrary to neurons from the central nervous system, DRG neuronal culture is associated with multiple challenges. One of the major obstacles in obtaining monolayers of DRG neurons, an absolute requirement for a reproducible and reliable analysis using the Seahorse XF24 analyzer, is their propensity to aggregate and detach from culture plates. Additionally, DRG neurons are sensitive to seeding density, culture media, coating substrate, and type of XF24 microplate plastic, all of which could contribute to detachment from the culture plate. These factors could potentially lead to an underestimation of cell number and significantly influence the interpretation of results. In the present work, we describe a comprehensive method that allows obtaining neuron-enriched cultures of primary embryonic (E15) rat DRG neurons that form monolayers of firmly attached cells at the optimum seeding density necessary for analysis of mitochondrial respiration with the Seahorse XF24 analyzer.

## Materials and Methods

### Materials

#### Chemicals

Ethylenediaminetetraacetic acid (EDTA), ethyleneglycoltetraacetic acid (EGTA), NaCl, sodium deoxycholate, boric acid, Triton X-100, sodium dodecyl sulfate (SDS), phenylmethylsulfonyl fluoride (PMSF), and phosphatase inhibitor cocktails 2 and 3 were purchased from Sigma-Aldrich (St. Louis, MO, USA). Complete EDTA-free protease inhibitor cocktail tablets were purchased from Roche Diagnostics (Indianapolis, IN, USA). Tris-HCl was purchased from Bio-Rad (Hercules, CA, USA). XF calibrant solution, dimethyl sulfoxide (DMSO), oligomycin A, carbonyl cyanide 4-(trifluoromethoxy)phenylhydrazone (FCCP), rotenone, and antimycin A were purchased from Seahorse Bioscience (Billerica, MA, USA).

#### Culture reagents

5-Fluoro-2′-deoxyuridine (FUDR), 1-β-d-ribofuranosyluracil (uridine), and d-(+)-glucose were purchased from Sigma-Aldrich (St. Louis, MO, USA). Minimal Essential Medium (MEM), Leibovitz’s L-15 media, B-27, Neurobasal media, Dulbecco’s phosphate buffered saline (DPBS), l-glutamine, and Pen-Strep were purchased from Gibco Life Technologies by Invitrogen (Grand Island, NY, USA). Hank’s Buffered Salt Solution (HBSS) and sodium pyruvate were purchased from Cellgro by Mediatech (Manassas, VA, USA). Bovine calf serum (BCS) of US origin was purchased from Hyclone by Thermo Scientific (Logan, UT, USA). Trypsin was purchased from Worthington Biochemical (Lakewood, NJ, USA). Nerve growth factor (NGF) was purchased from Bioproducts for Sciences, a division of Harlan Sprague-Dawley (Indianapolis, IN, USA). Thy 1.1 (CD90) rat anti-mouse antibody was purchased from ProSpec-Tany TechnoGene Ltd. (Ness Ziona, Israel). XF assay medium was purchased from Seahorse Bioscience (Billerica, MA, USA).

#### Culture dishes

All polyethylene terephthalate and polystyrene 24-well Seahorse XF24 microplates and cartridges were purchased from Seahorse Bioscience (Billerica, MA, USA). All other culture dishes (standard 96-well microplates, 60 mm dishes) were purchased from BD Biosciences (San Jose, CA, USA).

#### Animals

The Mayo Clinic Institutional Animal Care and Use Committee (IACUC) approved all animal studies involving DRG neurons extracted from wild-type embryonic day 15 (E15) Sprague-Dawley rats (Harlan Sprague-Dawley, Madison, WI, USA).

### Methods

#### Microplate substrate coating and plastic

It should be noted that the surface area of each well of 24-well Seahorse XF24 microplates is identical to that of standard 96-well microplates (0.32 cm^2^). The following coating substrates were tested on XF24 microplates: rat tail collagen Type I (5–50% in sterile deionized water; 30–40 μl/well) was purchased from BD Biosciences (Bedford, MA, USA); natural mouse laminin (2 μg/ml in sterile DPBS; 30–40 μl/well) was purchased from Gibco Life Technologies by Invitrogen (Grand Island, NY, USA); poly-l-lysine (0.1 mg/ml in sterile deionized water; 500 μl/well) and poly-l-ornithine (0.5 mg/ml in sterile deionized water or borate buffer [pH 8.4]; 500 μl/well) were purchased from Sigma-Aldrich (St. Louis, MO, USA).

Coating procedures used for Seahorse XF24 and standard 96-well microplates were as follows: microplates covered with poly-lysine were incubated overnight at 37°C, 5% CO_2_, and 95% humidity. The substrate solution was then removed, and the microplates were dried without rinsing under a sterile laminar flow hood. Poly-ornithine (dissolved in borate buffer) was added to the microplates, which were covered with foil and incubated overnight at room temperature (RT) under a sterile laminar flow hood. The substrate solution was then removed, wells were rinsed twice with sterile deionized water, and the microplates were dried under a sterile laminar flow hood. Laminin was only used in a combination with poly-lysine or poly-ornithine. Laminin was combined with poly-lysine and co-incubated according to the above poly-lysine coating procedure. Following poly-ornithine coating, laminin was applied to each well of the microplate and allowed to dry (unrinsed) at RT under a sterile laminar flow hood. Collagen was applied alone or in a combination with either poly-lysine or poly-ornithine. When applied alone, collagen was added to each microplate well and allowed to dry (unrinsed) at RT under a sterile laminar flow hood. When used in combination, collagen was added following the above poly-lysine or poly-ornithine coating procedures, and allowed to dry (unrinsed) at RT under a sterile laminar flow hood. Application of collagen and laminin involved adding enough solution to cover the bottom of the microplate well, while avoiding excessive capillary action from well edges, which can cause uneven distribution of these particular substrates. In addition, DRG attachment and distribution was evaluated using polystyrene and polyethylene terephthalate XF24 microplates to test the effect(s) of variations in the physical properties of each plastic on attachment and distribution of DRG neuronal culture.

#### Dissociated DRG neuronal culture and plating

Dissociated DRG neurons were isolated as described previously (Wood, 1976; McDonald et al., 2005), with modifications described below. Whole DRG explants were removed by aseptic microdissection from E15 Sprague-Dawley rats and dissociated with 0.25% trypsin in HBSS for 30 min, followed by mechanical dissociation by glass Pasteur pipet, flamed to reduce opening to <200 μm. Cells were re-suspended at 2 ml per 10 spines in 15% serum AN_2_ media (MEM, 15% BCS, 39 mM glucose, 1.2 mM l-glutamine, and fresh 10 ng/ml NGF). Cells were pre-plated on either uncoated or Thy 1.1-coated culture dishes (1:100 in L-15 or MEM for at least 4 h at RT or overnight at 4°C) at 10 embryo spines per 60 mm dish for 1.5–2 h in a humidified incubator at 37°C and 5% CO_2_. Pre-plating was used to remove non-neuronal cells in order to increase accuracy of cell counting and seeding, as well as to reduce the amount of time cells would spend in anti-mitotic media. The amount of spines allotted per 60 mm dish was optimized to avoid over-crowding, while the pre-plating incubation time was optimized to allow support cells to adhere but not DRG neurons.

Following pre-plating, the cell suspension was transferred to a 15 ml tube, the dishes were gently rinsed with 15% serum AN_2_ medium, and the rinse added to the cell suspension. Cells were concentrated in a Beckman TJ-6 centrifuge (Fullerton, CA, USA) at 3,000 rpm for 5 min at RT, and re-suspended in an appropriate volume of 15% serum AN_2_ in order to seed each well of a 24-well Seahorse XF24 microplate or standard 96-well microplates at 25,000–100,000 cells/well using a volume of 150 μl or less. After seeding, plates remained at RT for 45 min to 1 h before bringing the total volume of each well to 500 μl (XF24 microplates) or 200 μl (96-well microplates) with 15% serum AN_2_, followed by overnight incubation at 37°C, 5% CO_2_, and 95% humidity. Leaving plates at RT for up to 1 h promotes even distribution and adhesion of cells and avoids edge-effect within wells and on the plate as a whole (Burt et al., 1979; Oliver et al., 1981; Lundholt et al., 2003). Cultures were then treated with 20 μM FUDR and 20 μM uridine in 15% serum AN_2_ for 3–5 days with media changed every other day. This method eliminates ∼99% of non-neuronal supporting cells. DRG neuron-enriched cultures were incubated in either 15% serum AN_2_ without FUDR/uridine or Neurobasal media supplemented with B-27 (a serum substitute),1.2 mM l-glutamine, fresh 10 ng/ml NGF, and Pen-Strep (without FUDR/uridine) for 24 h prior to experiments (see Optimized Step-by-Step Protocol in Appendix).

#### Cell proliferation assay

The Promega CellTiter 96^®^ AQ_ueous_ One Solution Cell Proliferation Assay (Madison, WI, USA), or 3-(4,5-dimethylthiazol-2-yl)-5-(3-carboxymethoxyphenyl)-2-(4-sulfophenyl)-2H-tetrazolium, inner salt (MTS) assay, was completed according to the manufacturer’s instructions. The amount of formazan product measured at an absorbance of 490 nm is directly proportional to the number of living cells in culture, and thereby provides a method to determine the percentage of cell death that occurs from original plating to the time of assay. After plating DRG neurons onto poly-lysine-coated 96-well microplates, cells were maintained in 15% serum AN_2_ media (with FUDR/uridine) for 3 days and then placed in Neurobasal media (without Pen-Strep or FUDR/uridine) for 3 days. The MTS assay was completed at 7 days in culture (DIC). DRG neuron suspensions from each pre-plating condition (uncoated or Thy 1.1-coated 60 mm dishes) were seeded at densities of 25,000; 50,000; 75,000; and 100,000 cells/well of a standard 96-well microplate. PC12 cells were used to create a standard curve to calculate DRG neuronal densities in all wells of the 96-well microplates. PC12 cells were plated 3 h prior to the assay and seeded in triplicate at 0; 6,250; 12,500; 25,000; 50,000; 100,000; 150,000; and 200,000 cells/well of a standard 96-well microplates.

#### Seahorse XF24 mitochondrial stress analysis

Uptake and secretion of metabolic end-products such as oxygen and protons to and from the extracellular milieu allows the Seahorse XF24 Extracellular Flux analyzer to conduct real-time measurements of oxygen consumption and extracellular acidification of the surrounding microenvironment using solid-state fluorescent oxygen and pH biosensors coupled to a fiber-optic waveguide (Wu et al., 2007). The consumption and flux of oxygen and protons causes rapid and measurable changes in oxygen tension and pH within a transient microchamber created by the sensor cartridge and well plate while in the measurement position (see Figure 1 of Wu et al., 2007). Mitochondrial complex inhibitors are preloaded in the injection ports and are subsequently injected into the well media. After a short period of mixing, oxygen consumption rate (OCR) and extracellular acidification rate (ECAR) measurements are made using highly sensitive photodetectors specific for the excitation and emission of oxygen (532/650 nm) and protons (470/530 nm; Wu et al., 2007). Additional details relating to the Seahorse XF24 analyzer can also be found at http://www.seahorsebio.com/.

Prior to the Seahorse XF24 mitochondrial stress analysis, representative wells from each substrate and DRG neuron seeding density were imaged on a Zeiss Axiovert 35 inverted light microscope (Thornwood, NY, USA) using 5× phase objective lens at 1 DIC and on the day of flux analysis (all wells were observed for continuity in plating, cell density, and distribution). The Seahorse XF24 mitochondrial stress test was conducted as described previously, with exceptions noted (Wu et al., 2007; Ferrick et al., 2008). Specifically, DRG neurons on XF24 microplates were rinsed once and re-suspended in 500–675 μl of XF assay buffer (DMEM without NaHCO_3_, 7 mg/ml d-glucose, 2 mM glutamax; pH 7.4), then equilibrated for 1 h at 37°C in a non-CO_2_ incubator. All medium and solutions of mitochondrial complex inhibitors were adjusted to pH 7.4 on the day of assay. Following three baseline measurements of OCR and ECAR, mitochondrial complex inhibitors were sequentially injected into each well. Three OCR and ECAR readings were taken after addition of each inhibitor and before automated injection of the subsequent inhibitor. Mitochondrial complex inhibitors, in order of injection, included oligomycin (1.5 μM) to inhibit complex V (i.e., ATP synthase), FCCP (0.75 μM) to uncouple the proton gradient, antimycin A (1.0 μM) to inhibit complex III, and rotenone (1.0 μM) to inhibit complex I. Optimization of cell density and working concentration titers for each individual inhibitor was completed prior to the Seahorse XF24 mitochondrial stress analysis according to the Seahorse XF24 User’s Manual (Seahorse Bioscience, Billerica, MA, USA). OCR and ECAR were automatically calculated, recorded, and plotted by Seahorse XF24 software version 1.8 (Seahorse Bioscience, Billerica, MA, USA). At the end of each assay, cells were washed once with an excess of RT DPBS, lysed with ice-cold RIPA buffer (0.15 M NaCl, 1 mM EDTA [pH 8], 1 mM EGTA, 0.5% sodium deoxycholate, 0.1% SDS, 1% Triton X-100, 50 mM Tris-HCl [pH 8], and protease and phosphatase inhibitor cocktails), and the protein content estimated by Bio-Rad D_c_ protein assay (Bio-Rad, Hercules, CA, USA) using a Molecular Devices Softmax M3 microplate reader (Sunnyvale, CA, USA). Data was normalized for total protein content per well.

#### Statistical analysis

Normalized Seahorse XF24 measurements were averaged per density group per plate via Seahorse XF24 software version 1.8 (Seahorse Bioscience, Billerica, MA, USA) as mean ± SEM.

## Results

### Cell proliferation assay

**Figure 1:**
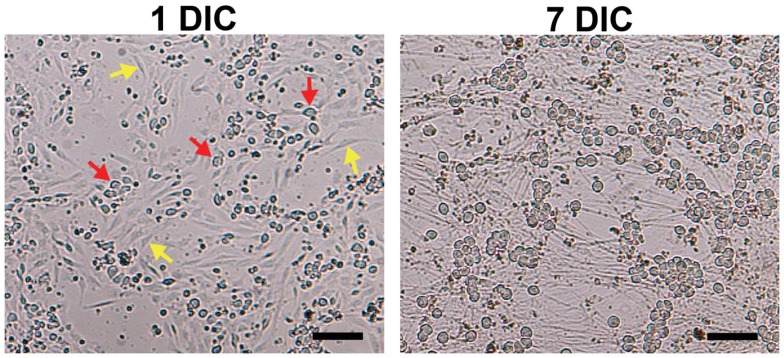
**Elimination of non-neuronal cells in DRG neuronal cultures**. Although Thy 1.1 pre-plating alone did not completely remove non-neuronal cells (1 DIC, yellow arrows; red arrows, DRG neurons), it significantly reduced the amount of time DRG neurons spent in anti-mitotic media. Dissociated DRG neurons seeded at 50,000 cells/well in a 24-well Seahorse XF24 polystyrene microplate at 1 and 7 DIC at 5× phase objective. Scale bar: 200 μm.

Pre-plating was used to remove non-neuronal cells in order to increase accuracy of cell counting and seeding, as well as to reduce the amount of time cells would spend in anti-mitotic media. The amount of spines allotted per 60 mm dish was optimized to avoid over-crowding, while the pre-plating incubation time was optimized to allow support cells to adhere but not DRG neurons. Results of the MTS assay demonstrated that pre-plating DRG neuron suspensions for 1.5–2 h on dishes pre-coated with Thy 1.1, followed by re-plating of unattached cells onto 96-well microplates and treating with FUDR/uridine for 3–5 days eliminates ∼99% of support cells from culture (Figure 1; Table 1). Thy 1.1 pre-plating also significantly improved the accuracy of DRG neuron counting using Trypan blue exclusion. However, the length of FUDR/uridine treatment was dependent on initial seeding density and plate surface area. Live cell counts based on absorbance intensity demonstrated an average of 38% cell loss in those wells that contained neuronal suspensions originally pre-plated on Thy 1.1-coated dishes, which is attributed to the removal of support cells. The absorbance intensity in wells containing neuronal suspensions pre-plated on uncoated 60 mm plates increased up to 61%, suggesting inefficient removal and proliferation of support cells (Table 1). The continued presence of non-neuronal cells despite FUDR/uridine treatment in wells that contained cells originally pre-plated on uncoated dishes was confirmed by daily monitoring cell growth and morphology in culture up to the day of MTS assay (7 DIC). Additionally, data demonstrate that the optimum lowest DRG neuron seeding density for Seahorse XF24 analysis was 50,000 cells/well, as DRG neurons could not survive seeding densities at or below 25,000 cells regardless of pre-plating method.

**Table 1:** **Efficiency of pre-plating DRG neuron suspensions on coated and uncoated 60 mm culture dishes at support cell removal**.

Pre-plating coating^†^	Seeding density (cells/well)^†^	% Cell loss*	% Cell gain/loss*	Avg% cell gain/loss**
Anti-Thy 1.1	25,000	−90.02**		−38.76
	50,000	−38.27		
	75,000	−42.08		
	100,000	−35.93		
Uncoated	25,000		−35.56**	+61.77
	50,000		+87.85	
	75,000		+64.99	
	100,000		+32.48	

*^†^After a 1.5–2 h pre-plating incubation, cell suspensions were collected and seeded onto standard 96-well microplates, where the surface area of each well is identical to that of Seahorse XF24 microplates (0.32 cm^2^/well)*.

* Gain/loss based on the number of live cells calculated at 7 DIC via Promega CellTiter 96^®^ AQ_ueous_ One Solution Cell Proliferation Assay (i.e., MTS assay).

**Average (Avg)% gain/loss excludes the 25,000 seeding density group, as values from both pre-plating methods were statistical outliers via rejection quotient *Q* with ≥95% confidence. Thus, seeding XF24 microplates at 25,000 cells/well was not a viable test group.

### Substrate coating

**Figure 2:**
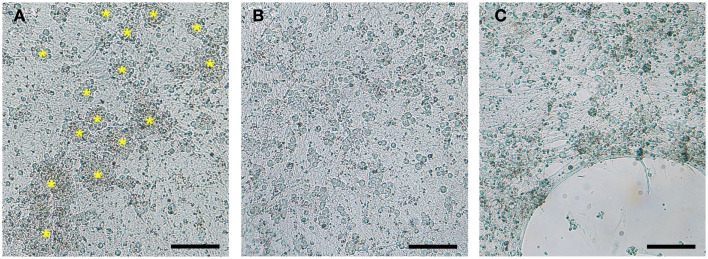
**Optimization of coating substrates for DRG neuronal culture**. **(A)** 24-well Seahorse XF24 microplates coated with collagen promoted aggregation and clumping of DRG neurons (yellow asterisks). **(B)** Poly-lysine, poly-ornithine, and the optimal substrate combination of poly-lysine + 10% collagen provided equally satisfactory DRG neuron monolayers. **(C)** Detachment of DRG neurons cultured on poly-lysine and poly-ornithine alone or in combination with laminin observed after 6 DIC. DRG neurons seeded at 50,000 cells/well in a XF24 polystyrene microplate imaged at 7 DIC with a 5× phase objective. Scale bar: 200 μm.

Seahorse XF24 microplates coated with ≥40% collagen maintained healthy and well-attached DRG neurons at 50,000 cells/well for up to 10 DIC. In addition, DRG neurons showed no evidence of detachment after addition of all four mitochondrial complex inhibitors during the Seahorse XF24 mitochondrial stress assay (∼2 h) regardless of XF24 microplate plastic type. DRG neurons seeded at densities below 50,000 cells/well tended to lift by 8–9 DIC on both XF24 microplate plastic types coated with collagen at concentrations ≤30%. Cells also exhibited significant detachment after addition of just one mitochondrial complex inhibitor. Conditions were not improved by UV cross-linking of the collagen coating on microplates prior to cell culture (known to improve durability and life of collagen coatings; Caruso and Dunn, 2005). Despite the strength of collagen as a coating substrate, DRG neurons tended to aggregate and clump at all concentrations tested (Figure 2A).

Poly-l-lysine and poly-l-ornithine substrates provided equally satisfactory DRG neuron monolayers on both XF24 microplate plastic types (Figure 2B). However, DRG neurons plated on these substrates at densities below 75,000 cells/well must be analyzed within 6 DIC to avoid significant neuronal detachment before the Seahorse XF24 assay can even be attempted, regardless of XF24 microplate plastic type (Figure 2C). Combinations of laminin and poly-lysine or poly-ornithine did not improve the adhesive qualities of either substrate beyond that which they already possessed on either XF24 microplate plastic type.

**Figure 3:**
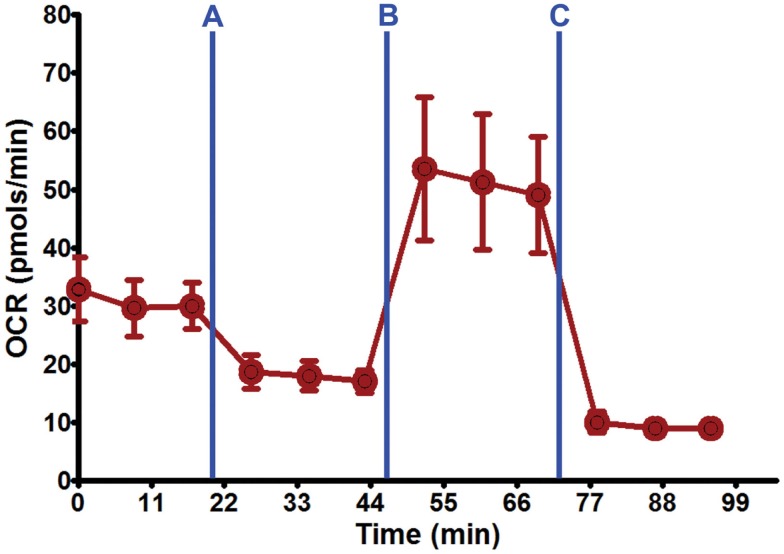
**Seahorse XF24 mitochondrial stress analysis**. Using the optimal seeding density (50,000 cells/well) and coating substrate combination (poly-lysine + 10% collagen) on 24-well Seahorse XF24 polystyrene microplate, well-adhered, and dispersed DRG neuronal cultures were maintained throughout the culture process and Seahorse XF24 analysis (7 DIC). Each data point is presented as mean ± SEM. Injection series: **(A)** Oligomycin (1.5 μM); **(B)** FCCP (0.75 μM); **(C)** rotenone (1.0 μM) and antimycin A (1.0 μM).

Optimal DRG neuron culture was achieved when 10% collagen was added to poly-lysine and poly-ornithine substrates. This combination produced firmly attached, non-aggregated DRG neuronal cultures at 50,000 cells/well on both XF24 microplate plastic types (Figure 2B; Table 2) that can withstand the Seahorse XF24 mitochondrial stress analysis (∼2 h) without lifting at 8 DIC (Figure 3). This allowed for subsequent estimation of protein concentration for normalization and further data analysis. Collagen concentrations ≥20% used in combination with poly-lysine or poly-ornithine increased the tendency of DRG neurons to aggregate and clump.

**Table 2:** **Optimal conditions for plating DRG neurons for Seahorse XF24 Extracellular Flux analysis^§^**.

Pre-plating method^†^	Growth media	Coating substrate	XF24 microplate plastic type	Seeding density
Coating: anti-Thy 1.1 Dishes: standard 60 mm, plastic Incubation: 1.5–2 h; 37°C, 5% CO_2_, 95% humidity	15% serum AN_2_ media	Poly-l-lysine (0.1 mg/ml) + collagen (10%)	Polyethylene terephthalate or polystyrene	50,000 cells/well*

*^§^See the detailed step-by-step protocol for culturing embryonic DRG neurons*.

*^†^Following incubation, cell suspensions were collected, counted, and seeded onto Seahorse XF24 microplates*.

*Seeding density per well of an XF24 microplate.

### Culture medium

Within 24 h after the addition of supplemented Neurobasal media, DRG neurons migrated into large clumps and began to rapidly detach and die, regardless of the coating substrate and XF24 microplate plastic (Figures 2A,C). By contrast, 15% serum AN_2_ medium allowed preservation of healthy, non-aggregated DRG neuronal cultures (Figure 2B). These data suggest 15% serum-supplemented AN_2_ medium is optimal for culture of healthy DRG neuron cultures at the optimum lowest seeding density (50,000 cells/well) for Seahorse XF24 analysis.

### Seahorse XF24 cell density titration

**Figure 4:**
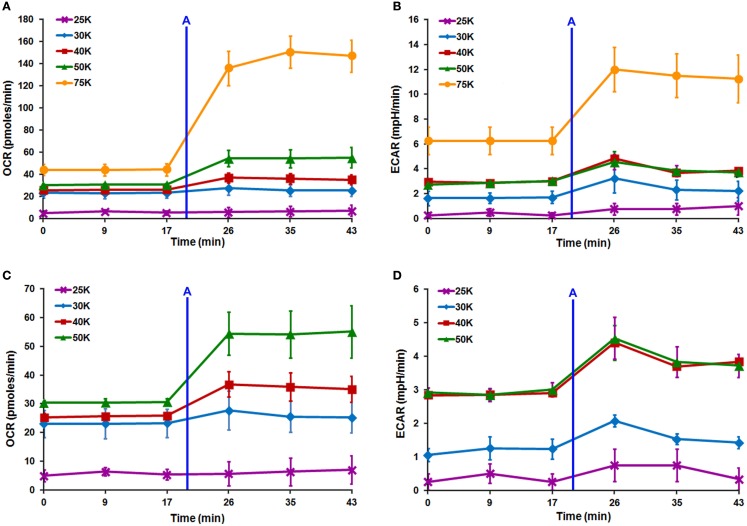
**Seahorse XF24 optimization of DRG neuron seeding density**. Titration of DRG neuron seeding density with the XF24 platform maximizes increases and decreases in OCR and ECAR. Linear increases are seen in both **(A)** OCR and **(B)** ECAR spectra with increasing cell number. **(A–D)** XF24 microplates seeded at 25,000 cells/well (purple line) produced negligible OCR and ECAR due to detachment either before or during analysis. **(D)** ECAR values plateau between 40,000 (red line) and 50,000 (green line) cells/well indicating the optimal seeding density for Seahorse XF24 analysis is likely 40,000 cells/well. After Seahorse XF24 mitochondrial stress analysis, XF24 microplates seeded at 40,000 cells/well showed minor detachment, thus, 50,000 cells/well was selected as the optimal density. Each data point is presented as mean ± SEM. All readings completed on polystyrene XF24 microplates pre-coated with 0.1 mg/ml poly-lysine + 10% collagen. Injection **(A)** FCCP (0.5 μM).

Seahorse XF24 cell density titration was completed to determine the optimum lowest DRG neuronal seeding density that produced optimal OCR and ECAR readouts. Increasing cell density resulted in linear increases in both OCR and ECAR (Figures 4A–D). When neurons were seeded at 25,000 cells/well, OCR and ECAR values were negligible due to the detachment of cells either before or during the analysis (Figures 4A–D). ECAR spectra show that the increase in acidification rate with increasing cell number does not significantly change between 40,000 and 50,000 cells/well (Figure 4D). This indicated that 40,000 cells/well was the optimum lowest seeding density where optimal increases and decreases in both OCR and ECAR were obtained within the linear response range. However, following initial Seahorse XF24 mitochondrial stress analysis, neurons seeded at 40,000 cells/well showed minor detachment at well edges, whereas those seeded at 50,000 cells/well did not. Therefore, 50,000 cells/well was confirmed as the optimum lowest DRG neuronal seeding density for Seahorse XF24 analysis.

### Microplate temperature and plastic type

**Figure 5:**
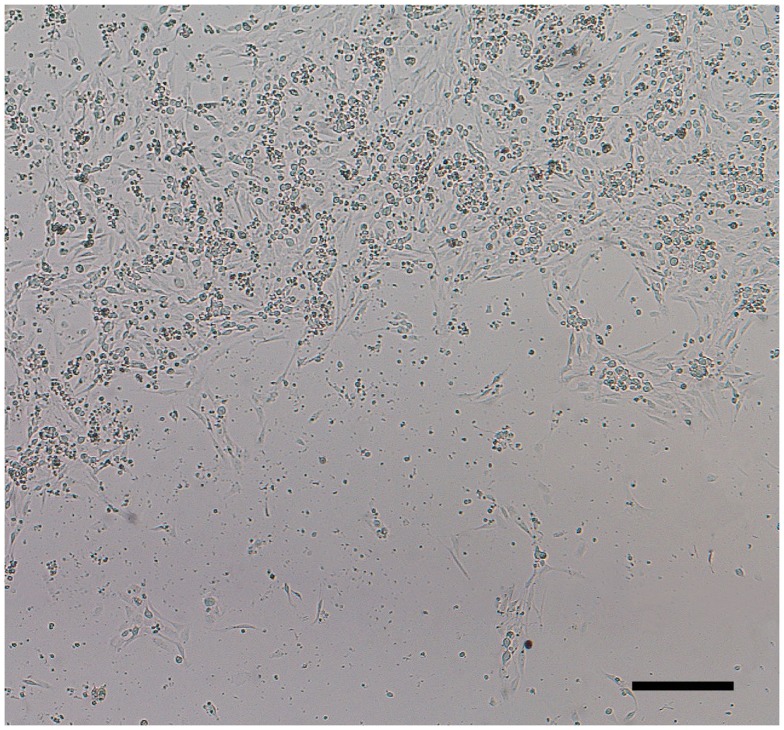
**“Edge-effect.”** DRG neurons cultured in 24-well Seahorse XF24 microplates at RT display uneven distribution when placed directly in a humidified incubator at 37°C and 5% CO_2_. This “edge-effect” is caused by a thermal gradient between the periphery and center of each well. DRG neuronal culture at 1 DIC seeded at 50,000 cells/well of an XF24 polystyrene microplate at 5× phase objective. Scale bar: 200 μm.

Results demonstrated that when using the optimal substrate (poly-lysine + 10% collagen) and seeding density (50,000 cells/well), DRG neuron attachment and growth were similar on both polyethylene terephthalate and polystyrene Seahorse XF24 microplates. However, it was found that regardless of substrate and XF24 microplate plastic type, DRG neurons were particularly sensitive to temperature. “Edge-effect” within each well and on the microplate as a whole was observed when newly seeded DRG neuronal cultures at RT were directly transferred to a humidified incubator at 37°C and 5% CO_2_ (Figure 5). This effect did not correct itself with time. “Edge-effect” was prevented when cultures were left to equilibrate at RT for at least 45 min to 1 h before being placed in a 37°C, 5% CO_2_ humidified incubator.

## Discussion

The major requirements for obtaining reproducible and reliable data with the Seahorse XF24 analyzer is working with a consistently uniform population of target cells that are evenly distributed and firmly attached to the culture dish. We first optimized the conditions that would allow obtaining enriched cultures of primary rat DRG neurons using 24-well Seahorse XF24 microplates. A significant obstacle in obtaining pure DRG neuronal cultures in dishes with diameter less than 35 mm is the presence of highly proliferative support cells. While a previous study by Delree et al. (1989) demonstrated that dissociated DRG neurons can be purified by Percoll gradient centrifugation, this protocol is time-consuming, uses abrasive enzymatic dissociative treatment that can contribute to neuronal cell loss, and does not allow for complete removal of Percoll reagent after plating. Alternatively, many studies have shown the effectiveness and ease of use of FUDR/uridine treatment that applied at low concentrations and for the period of up to 5 days does not cause cellular toxicity (Wallace and Johnson, 1989; Fex Svenningsen et al., 2003). Therefore, we examined different pre-plating conditions in conjunction with FUDR/uridine anti-mitotic treatment.

Pre-plating cells onto uncoated or coated culture dishes is a relatively fast, effective, and cell stress-free technique commonly used to separate cells based on differential adhesive properties (Banker and Goslin, 1998; Yang et al., 2010). The MTS assay was used to determine the efficacy of pre-plating on uncoated and Thy 1.1-coated 60 mm plastic culture dishes to remove non-neuronal cells prior to culturing DRG neurons on dishes with a surface area of 0.32 cm^2^ (Seahorse XF24 and standard 96-well microplates). Results of the MTS assay demonstrated that pre-plating DRG neuron suspensions for on Thy 1.1 pre-coated dishes, followed by re-plating unattached cell suspensions onto microplates and treating with FUDR/uridine for 3–5 days eliminates ∼99% of support cells from culture (Figure 1; Table 1). Although Thy 1.1 pre-plating alone did not remove 100% of non-neuronal cells, it did significantly reduce the amount of time DRG neurons needed to spend in anti-mitotic media (Table 1). Furthermore, these data suggested that the optimal lowest seeding density for Seahorse XF24 analysis was between 50,000 and 100,000 cells/well.

In order to further validate MTS assay results, multiple DRG neuronal densities were assessed via Seahorse XF24 analyzer to determine the lowest optimal cell number that would produce optimal OCR and ECAR values. Following injection of low dose of FCCP after three baseline measurements, linear increases in both OCR and ECAR were observed with increasing cell density (Figures 4A–D). Further examination of the ECAR spectra demonstrated that the increase in ECAR with increasing cell number began to plateau between 40,000 and 50,000 cells/well (Figure 4D). According to the Seahorse XF24 User’s Manual, this result indicated that the optimum lowest DRG neuronal seeding density was 40,000 cells/well, as it produced an optimal increase and decrease in both OCR and ECAR values and was within the linear response range for DRG neurons. However, following preliminary Seahorse XF24 mitochondrial stress analysis, wherein all four mitochondrial complex inhibitors were sequentially injected within a ∼2 h time period, XF24 microplates seeded at 40,000 cells/well showed minor detachment at the edges of each well, whereas those seeded at 50,000 cells/well did not. This detachment was exacerbated upon rinsing with PBS while preparing cells for protein estimation, thereby potentially introducing error into post-normalization analysis of the data. Therefore, 50,000 cells/well was selected as the optimum lowest DRG neuronal seeding density for Seahorse XF24 analysis.

Having determined the optimum lowest DRG neuron seeding density for Seahorse XF24 analysis, we then optimized the coating substrate, which would extend the life, health, and dispersion of DRG neurons in culture. Plating and maintaining monolayers of neuron-enriched DRG cultures is essential to accurately measure their metabolic state using the Seahorse XF24 analyzer. The durability of coating substrates is crucial to applications in which DRG neurons are to be treated with experimental drugs, which cannot occur until the cells have been established in culture and resting in anti-mitotic-free media for at least 24 h (a minimum 4–5 DIC period). The simple increase of DIC with experimental drug treatments lasting more than 12 h could lead to cell lifting before Seahorse XF24 analysis can be completed. Moreover, the experimental drug itself could weaken the culture and cause premature DRG neuron detachment.

We determined that combining a positively charged coating substrate (i.e., poly-lysine) with the extracellular matrix (ECM) coating molecule collagen (10%) produced firmly attached, non-aggregated DRG neuronal cultures that can withstand the Seahorse XF24 mitochondrial stress analysis without lifting (at 8 DIC; Table 2). The need for an ECM-based coating substrate to increase DRG neuron adhesion has been well documented, establishing collagen-based substrates as optimal for the culture of primary DRG neurons (Letourneau, 1975; Gundersen and Barrett, 1984; Gundersen, 1988; Sango et al., 1993). Although, the presence of the positive charge afforded by poly-lysine and poly-ornithine is known to be sufficient for dissociated DRG neuronal cultures (Letourneau, 1975; Gundersen and Barrett, 1984; Gundersen, 1988; Delree et al., 1989), the current study demonstrates that at seeding densities below 75,000 cells/well on surface areas ≥0.32 cm^2^, these substrates alone are not able to maintain DRG neurons over 6 DIC.

This is not surprising *in lieu* of previous reports which show that the presence of trophic support factors other than NGF in media conditioned by glial cells is vital to the adhesion and survival of purified DRG neurons seeded at low density *in vitro* (Gundersen and Park, 1984; Delree et al., 1989). Our results also illustrate the speed and effectiveness with which Thy 1.1 pre-plating in combination with anti-mitotic treatment removes support cells, as this procedure precluded either the time required to condition media and/or the presence of enough glial cells with which to effectively condition the media. Furthermore, since the mere presence of support cells would provide a surface with which to securely attach DRG neurons (Letourneau, 1975; Gundersen and Barrett, 1984; Fallon, 1985), their absence, in the case of poly-lysine and poly-ornithine substrates, would leave DRG neuron attachment at the mercy of a single positive charge.

To ensure the healthiest cultures possible for analysis with the Seahorse XF24 analyzer, we next evaluated the best culture medium in which to grow embryonic DRG neurons at 50,000 cells/well on an XF24 microplate. The most commonly used medias to culture neurons are Neurobasal media (Banker and Goslin, 1998) and 15% serum AN_2_. Neurobasal medium was specifically designed to maintain healthy neuronal cultures while promoting removal of non-neuronal cells (Brewer et al., 1993). However, it was found that supplemented Neurobasal media caused DRG neurons to migrate into large clumps and rapidly detach (Figures 2A,C), while 15% serum AN_2_ medium maintained healthy, non-aggregated DRG neuronal cultures (Figure 2B). Thus, 15% serum-supplemented AN_2_ medium was found to be optimal for maintaining healthy DRG neuron cultures at the optimum lowest seeding density for Seahorse XF24 analysis (50,000 cells/well).

Finally, we determined that DRG neurons could be plated and maintained equally well on both polyethylene terephthalate and polystyrene plastic XF24 microplates, assuming seeding number, media, and coating substrates are optimized. Furthermore, DRG neurons were particularly sensitive to temperature. Studies have shown that when microplate cultures are transferred from RT to 37°C, a thermal gradient develops between the periphery and center of each well, as well as between the peripheral and center wells of the plate producing an attachment pattern known as the “edge-effect” (Figure 5) that does not correct itself with time (Burt et al., 1979; Oliver et al., 1981; Lundholt et al., 2003). “Edge-effect” can be eliminated by allowing newly seeded DRG neuronal cultures to stay at RT for at least 45 min to 1 h before being transferred to a humidified incubator at 37°C and 5% CO_2_.

In conclusion, we have developed a comprehensive protocol that allows reproducible and reliable preparation of DRG neurons suitable for the analysis of mitochondrial respiration using the Seahorse XF24 analyzer. Optimal conditions are summarized in Table 2 and the optimized step-by-step protocol for culturing E15 DRG neurons for XF24 analysis can be found below. This method will be instrumental for research involved in the investigation of mitochondrial dysfunction underlying peripheral neuropathies, among other neurodegenerative diseases.

## Conflict of Interest Statement

The authors declare that the research was conducted in the absence of any commercial or financial relationships that could be construed as a potential conflict of interest.

## Appendix

### Optimized step-by-step protocol

All culture materials, equipment, and reagents should be used sterile unless otherwise indicated.

#### Day 0: culture plate preparation (day before dissection)

(1) Coat Seahorse XF24 microplates by adding 500 μl of poly-lysine to each well and incubate overnight in a humidified incubator at 37°C and 5% CO_2_.
  *Coat 3–4 microplates per litter when seeding at 50,000 cells/well*.
(2) Prepare anti-Thy 1.1 solution and add 2 ml per 60 mm dish and incubate:
  - Overnight at 4°C.
    – OR –
  - At room temperature (RT) under a sterile laminar flow hood for ≥4 h.
  *Coat two 60 mm plastic dishes per litter*.

#### Day 1: dissection and plating

(1) Remove poly-lysine and allow XF24 microplates to dry under a sterile laminar flow hood.
(2) Add 30–40 μl of 10% collagen to each well and let XF24 microplates dry under a sterile laminar flow hood.
  *This amount supplies enough solution to cover the well bottom, while avoiding excessive capillary action from well edges, which can cause uneven distribution of collagen as it dries*.
(3) Dissect and isolate E15 DRG whole explants as described previously (Wood, 1976; McDonald et al., 2005).
(4) Transfer all DRG dissected from one litter to a plastic 15 ml conical tube using a glass Pasteur pipette.
  *Wash the pipette with 15% serum AN_2_ to prevent DRG from sticking*.
(5) Centrifuge the conical tube at 800 rpm for 5 min at RT and discard supernatant.
(6) Add 1–2 ml of 0.25% trypsin solution to the conical tube and incubate for 30 min in a 37°C water bath with constant gentle agitation.
(7) Centrifuge the cell suspension at 800 rpm for 5–10 min at RT and discard supernatant.
(8) Re-suspend DRG in warm 15% serum AN_2_ at 2 ml per 10 embryo spines.
  *Do not exceed this volume (see step 10, Day 1)*.
(9) Flame a few glass Pasteur pipettes to reduce the opening to <200 μm and mechanically dissociate DRG until the suspension is completely homogenous.
  *Wash the pipette with 15% serum AN_2_ to prevent DRG from sticking*.
(10) Remove the anti-Thy 1.1 solution from the 60 mm plates, add 2 ml of the dissociated DRG suspension to each 60 mm plate, and incubate suspensions on plates for 1.5–2 h in a humidified incubator at 37°C and 5% CO_2_.
  *Do not add more or less than 2–2.5 ml per 60 mm plate. More interferes with cell settling and plate adhesion; less results in drying of plates during incubation*.
(11) After 1.5 h, examine plates under an inverted light microscope at 10× phase objective to note the efficiency of non-neuronal cell binding to the culture plate and determine whether additional incubation time is needed.
  *Do not incubate longer than 2.5 h; DRG neurons will begin to adhere and cultures may dry out*.
(12) Transfer cells in suspension from each 60 mm plate to a plastic 15 ml conical tube, *gently* rinse the 60 mm dish with 1–2 ml of warm 15% serum AN_2_, and add the rinse to the cell suspension.
  *Both Thy 1.1 plates from each litter can be combined*.
(13) Repeat homogenization of step 9, Day 1, if necessary.
(14) Count cells using Trypan blue exclusion and a hemacytometer.
  *Cell types can usually be differentiated as follows: DRG neurons are typically largest, round, and phase-bright; non-neuronal cells (i.e., fibroblasts and Schwann cells) are smaller, flat, non-spherical, and phase-bright*.
  - If cells need to be concentrated:
    - Centrifuge cells in the plastic 15 ml conical tube at 3,000 rpm for 10 min at RT and discard supernatant.
    - Re-suspend cells in an appropriate volume of warm 15% serum AN_2_ in order to seed each well of the XF24 microplate using a volume of 50–150 μl or less
    - Repeat homogenization of step 9, Day 1.
(15) Seed cells at 50,000 cell/well on a XF24 microplate (pre-coated with poly-lysine + 10% collagen) using a volume of 50–150 μl or less per well.
  *Do not add more or less than 50–150 μl/well. More solution interferes with cell settling and plate adhesion; less results in drying of plates during incubation in step 16, Day 1*.
(16) Incubate cells on XF24 microplates at RT under a laminar flow hood for 45 min to 1 h before bringing the total volume of each well to 500 μl with RT 15% serum AN_2_ media.
  *The total volume of each well is arbitrary, as long as there is enough media to keep cultures hydrated for 24 h (≥200 μl) and the volume is known*.
  *Leaving plates at RT for 45 min to 1 h promotes even distribution and adhesion of cells and avoids edge-effect within wells and on the plate as a whole*.
(17) Incubate DRG neuronal cultures overnight in a humidified incubator at 37°C and 5% CO_2_.

#### Day 2: media exchange

(1) Examine XF24 microplates under an inverted light microscope at 5–10× phase objective to note neuronal health, distribution, etc.
(2) Remove media from each well, leaving at least 50 μl of media to cover cells, and replace with 450 μl (or appropriate total volume desired) of warm 15% serum AN_2_ containing 20 μM FUDR and 20 μM Uridine.
  *The total volume of each well is arbitrary, as long as there is enough media to keep cultures hydrated for 24–48 h (≥200 μl) and the volume is known*.
(3) Continue incubation of cultures in a humidified incubator at 37°C and 5% CO_2_.

#### Day 3 – day of assay: DRG neuronal culture and maintenance

(1) Repeat step 2, Day 2, replacing media every other day with fresh warm 15% serum AN_2_ containing FUDR/uridine until ∼99% of the non-neuronal cells are eliminated.
  *DRG neurons should not spend more than 5 days in culture in FUDR/uridine-containing media, as it will become toxic*.
(2) Repeat step 2, Day 2, using fresh warm FUDR/uridine-*free* 15% serum AN_2_ media for at least 24 h before cells are treated with experimental drugs or analyzed with the Seahorse XF24 platform.

### Recipes

All culture materials, equipment, and reagents should be used sterile unless otherwise indicated.

#### 15% serum AN_2_ media ([final] provided)

(1) 15% bovine calf serum
(2) 1.2 mM l-glutamine
(3) 7 mg/ml d-(+)-glucose
(4) Dilute in MEM (+Earl’s salts)
(5) Aliquot and store at −20°C
  **Add NGF (10 ng/ml) fresh with each 15% AN_2_ media exchange*.

#### 2 mM FUDR (5-fluoro-2′-deoxyuridine) stock (10 ml)

(1) 4.924 mg FUDR
(2) Dilute to 10 ml in ddH_2_O
(3) Store covered at 4°C for up to 2 weeks
  **Add fresh to 15% AN_2_ with each media exchange at 1:100 dilution (20 μM [final])*.

#### 2 mM Uridine (1-β-d-ribofuranosyluracil) stock (10 ml)

(1) 4.884 mg uridine
(2) Dilute to 10 ml in ddH_2_O
(3) Store covered at 4°C for up to 2 weeks
  **Add fresh to 15% AN_2_ with each media exchange at 1:100 dilution (20 μM [final])*.

#### 0.25% Trypsin (10 ml)

(1) 0.025 g Trypsin (216 U/mg protein)
(2) Dilute in HBSS (Ca^2+^and Mg^2+^-free)
  **Always make fresh the day of use*.

#### Nerve growth factor

(1) Use at 10 ng/ml in 2 mg/ml cytochrome-*c* as a carrier protein in MEM.
(2) Aliquot and store at −80°C.

#### Poly-l-lysine hydrobromide (50 ml)

(1) 5 mg of poly-l-Lysine Hydrobromide.
(2) Dilute in ddH_2_O.
(3) Excess solution can be stored at −20°C, but avoid multiple freeze-thawing.

#### Thy 1.1 (CD90) rat anti-mouse monoclonal antibody solution

(1) Rehydrate anti-Thy 1.1 to 1 mg/ml in DPBS.
(2) Aliquot and store at −20°C.
(3) Dilute 1:100 in MEM or L-15.
  *Use of MEM or L-15 does not appear to effect outcome*.
  **Add fresh to 15% AN_2_ with each media exchange at 1:100 dilution (20 μM [final])*.

### Tips

– Estimate ∼30–40 DRG tissues dissected per embryo spine (dependent on dissection efficiency; always estimate low).
– One DRG tissue contains ∼1.0 × 10^4^total cells; ∼60% support cells, ∼40% DRG neurons.
– Estimate 10 embryos per litter.
